# Principal factors that determine the extension of detection range in molecular beacon aptamer/conjugated polyelectrolyte bioassays†
†Electronic supplementary information (ESI) available: Additional UV-vis, PL and CV data, equilibrium constant calculation for MBAs conformational change, and CD data. See DOI: 10.1039/c4sc03258f



**DOI:** 10.1039/c4sc03258f

**Published:** 2015-01-07

**Authors:** Ji-Eun Jeong, Boram Kim, Shinjae Woo, Sungu Hwang, Guillermo C. Bazan, Han Young Woo

**Affiliations:** a Department of Nanofusion Engineering , Department of Cogno-Mechatronics Engineering , Pusan National University , Miryang , Gyeongsangnam-do 627-706 , Republic of Korea . Email: hywoo@pusan.ac.kr; b Department of Nanomechatronics Engineering , Pusan National University , Miryang , Gyeongsangnam-do 627-706 , Republic of Korea; c Department of Chemistry & Biochemistry , University of California , Santa Barbara , California 93106 , USA . Email: bazan@chem.ucsb.edu

## Abstract

A strategy to extend the detection range of weakly-binding targets is reported that takes advantage of fluorescence resonance energy transfer (FRET)-based bioassays based on molecular beacon aptamers (MBAs) and cationic conjugated polyelectrolytes (CPEs). In comparison to other aptamer-target pairs, the aptamer-based adenosine triphosphate (ATP) detection assays are limited by the relatively weak binding between the two partners. In response, a series of MBAs were designed that have different stem stabilities while keeping the constant ATP-specific aptamer sequence in the loop part. The MBAs are labeled with a fluorophore and a quencher at both termini. In the absence of ATP, the hairpin MBAs can be opened by CPEs *via* a combination of electrostatic and hydrophobic interactions, showing a FRET-sensitized fluorophore signal. In the presence of ATP, the aptamer forms a G-quadruplex and the FRET signal decreases due to tighter contact between the fluorophore and quencher in the ATP/MBA/CPE triplex structure. The FRET-sensitized signal is inversely proportional to [ATP]. The extension of the detection range is determined by the competition between opening of the ATP/MBA G-quadruplex by CPEs and the composite influence by ATP/aptamer binding and the stem interactions. With increasing stem stability, the weak binding of ATP and its aptamer is successfully compensated to show the resistance to disruption by CPEs, resulting in a substantially broadened detection range (from millimolar up to nanomolar concentrations) and a remarkably improved limit of detection. From a general perspective, this strategy has the potential to be extended to other chemical- and biological-assays with low target binding affinity.

## Abstract 2

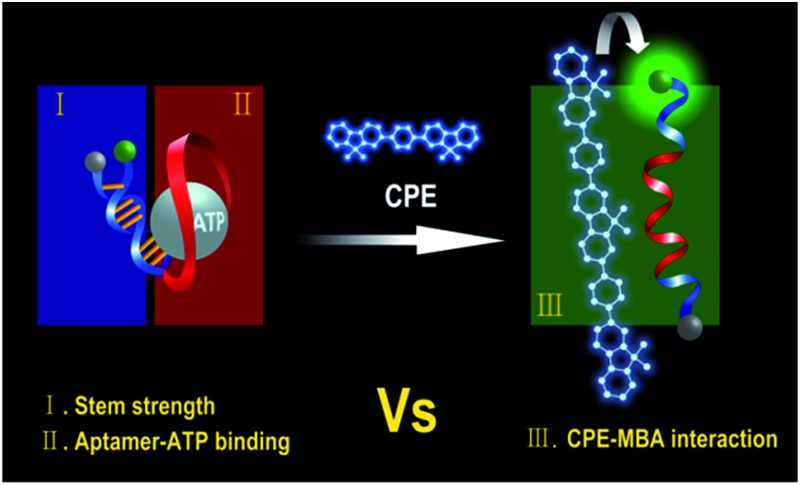
A new bioassay strategy based on the molecular beacon aptamer/conjugated polyelectrolyte demonstrates a fine-tuning of the detection range and limit of detection for weakly-binding targets.

## Introduction

Conjugated polyelectrolytes (CPEs) have emerged as attractive and versatile optical platforms for highly sensitive chemo- or biosensors^1–5^ that can detect a variety of targets, including metal ions, DNA, RNA and peptides.^6–11^ CPEs are described by a π-conjugated backbone with ionic pendant groups, which make them water-soluble and/or bio-compatible. A range of cationic and anionic CPEs have been synthesized for specific applications including bioimaging, bioelectronics, fluorescent probes and signaling mediation.^12–17^ Structural diversity allows the optical and electronic properties of CPEs to be adjustable and consequently takes advantage of their light harvesting properties.^18,19^

Molecular beacons (MB) are loop-stem hairpin-type signaling probes labeled with a fluorophore and a quencher at the two different termini of the stem. This structural feature in the hairpin structure leads to a substantially decreased photoluminescence (PL) emission by virtue of the fluorophore and quencher close proximity. In the presence of a complementary target, the MB probe undergoes a conformational change from hairpin to an open-chain structure, resulting in the recovery of PL from the fluorophore. Interesting strategies were recently reported to control the detection range of MB-based DNA assays by modulation of stem stability. For example, it has been reported that the stem length has a significant impact on the binding characteristics and hybridization kinetics of MBs. With increasing the stem length, target discrimination can be facilitated over a broader range of temperature but a decrease in the rate of MB/target hybridization was measured.^20^ Another approach for tuning the detection range has been demonstrated by controlling the stem stability of MB probes. Specifically, the detection range can be shifted toward higher target concentration with increases in the stability of the nonbinding hairpin structure.^21,22^ In an another electrochemical bioassay where a redox reporter-modified MB was attached to the electrode, hybridization of MB with a complementary target DNA induced stretching of the hairpin structure away from the electrode, giving rise to decreasing electron transfer from the reporter to electrode. This process was successfully modulated by changing the MB stem stability.^23^

**Scheme 1:**
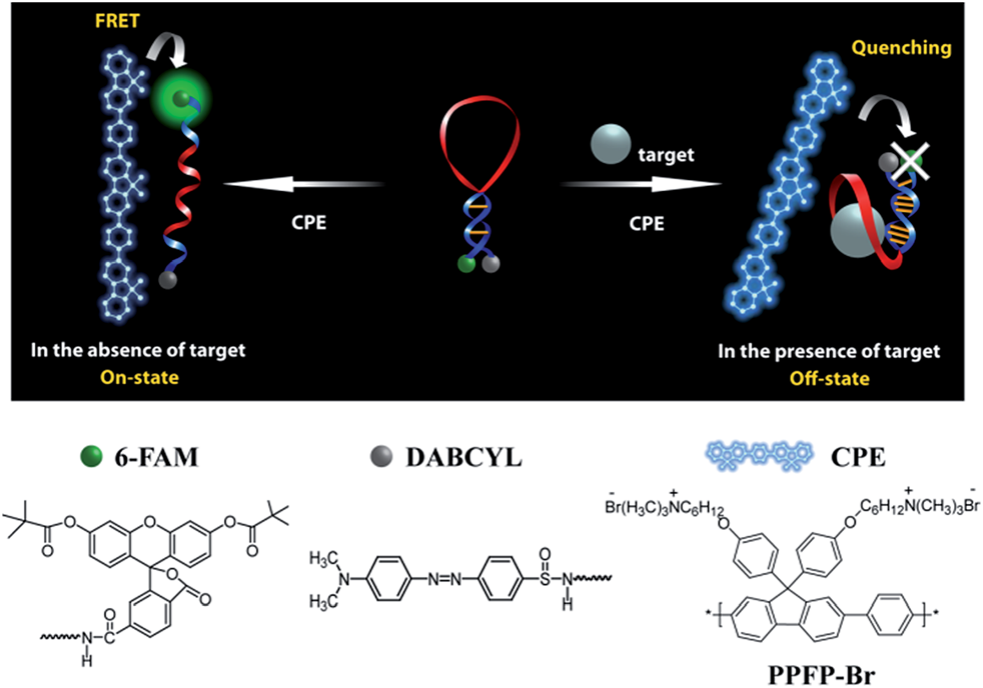
Schematic for MBA/CPE-based bioassay.

We recently demonstrated a highly sensitive and selective potassium (K^+^) detection assay with a limit of detection (LOD) of ∼1.5 nM based on a cationic poly(fluorene-co-phenylene) based CPE (**PPFP-Br**) and a oligonucleotide molecular beacon aptamer (MBA) containing a K^+^-specific aptamer base sequence in its loop part (Scheme 1).^24^ Aptamers are well-recognized target-specific biomolecules that may serve as recognition elements in biological assays, including those mediated by CPEs.^25–27^ They are defined as nucleic acid based ligands with high binding affinities toward broad range of molecules.^28,29^ In particular, single-stranded DNA aptamers with guanine (G)-rich sequences can bind to a given target and fold into a secondary G-quadruplex structure *via* intramolecular hydrogen-bonding interactions.^30–32^ The MBA is an aptamer-based MB containing a specific aptamer base sequence in the loop structure. Structural reorganization from hairpin to an open-chain conformation is induced by complexation with cationic CPEs.^33,34^ The open MBA structure in the presence of CPE shows a strong fluorescence resonance energy transfer (FRET)-induced fluorophore emission in the absence of K^+^ ions (on state). Upon addition of K^+^ ions, the MBAs prefer the G-quadruplex conformation, even in the presence of cationic CPEs, which leads to a decrease in PL (off state). Monitoring the CPE-sensitized fluorophore reporter emission intensity thus allows one to detect K^+^. This MBA/CPE based detection approach is operationally simple and may be extended to a wide range of target materials by modifying the aptamer sequence in the loop.

Based on the above studies, we describe in this contribution a novel strategy that combines biomolecular MBAs and a synthetic polymer, to tune the detection range for weakly binding target materials. We also provide the principal factors that govern the detection mechanisms. ATP was selected as the target material due to the relatively weak binding constant with its complementary aptamer. Specifically, a dissociation constant (*K*_d_) of μM–mM was reported previously for the ATP/aptamer complex.^35–39^ For comparison, a *K*_d_ value in the range of 2.68–450 nM was reported for K^+^/aptamer binding.^40^ Doxorubicin and human VEGF (vascular endothelial growth factor) can be captured by its aptamer with *K*_d_ = 600 nM and 50 pM, respectively,^41,42^ and the lysozyme aptamer binds to its target with *K*_d_ = 31 nM.^43^

A series of MBAs (MBA1–MBA5) were designed so that the differences in structure lead to different stem stabilities. The MBAs contain the same ATP-specific aptamer sequence within the loop substructure, while their stem interactions were fine-tuned by changing the number of guanine (G)-cytosine (C) triple hydrogen bonds. As described in detail, the extension of the detection range depends critically on the competition between opening of the ATP/MBA G-quadruplex by CPEs and the composite influence by ATP/aptamer binding and the stem interactions. With increasing stem stability (MBA1 → MBA5), the weak binding affinity of ATP and its aptamer was successfully compensated to show resistance to opening by CPEs, resulting in a significantly broadened detection range with remarkably improved LOD: from millimolar up to nanomolar concentrations.

## Results and discussions

### MBA/CPE-based detection scheme

**Scheme 2:**
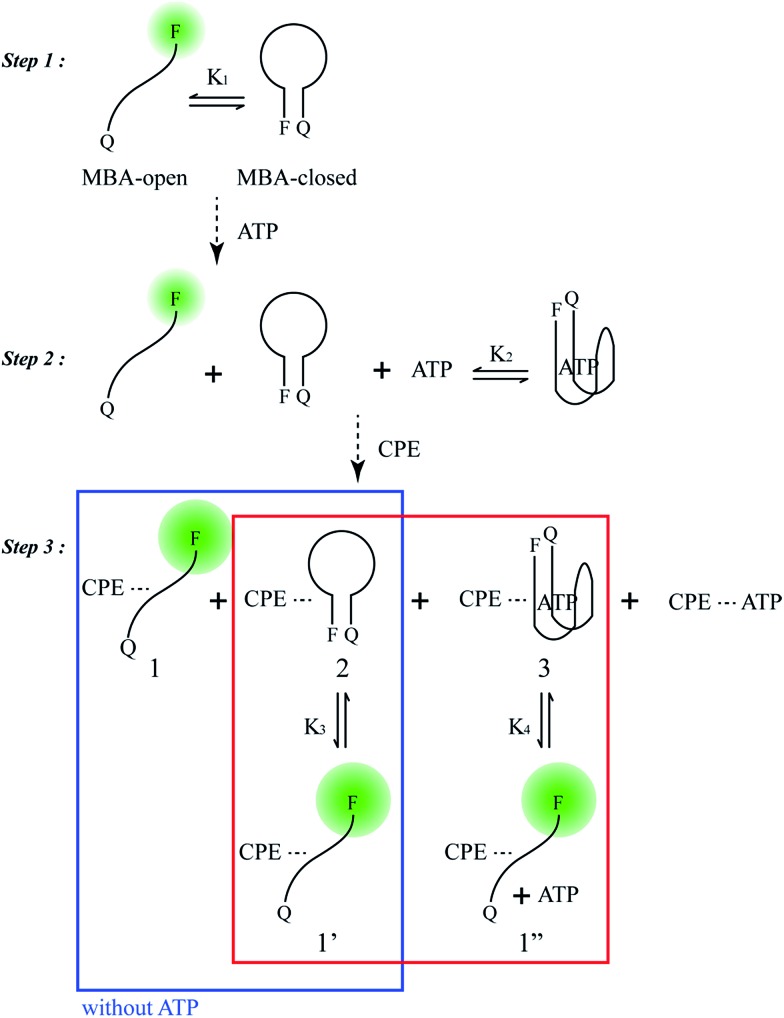
Principal participants in the ATP detection mechanism in the MBA/CPE assay.

Scheme 2 provides a sequence of relevant intramolecular conformational changes and intermolecular processes that form the basis of the MBA/CPE based FRET assay for ATP detection. The MBA structure contains a fluorophore (6-carboxyfluorescein (6-FAM), F) and a quencher (4-(4′-dimethylaminophenylazo)benzoic acid (DABCYL), Q) at the two different termini. At room temperature equilibrium, MBA exists primarily as a hairpin-type structure, MBA-closed (step 1, *K*_1_). The aptamer sequence of 5′ GGGG AGTA TTGC GGAG GA 3′, which has been reported to form a G-quadruplex specifically upon interaction with ATP,^35,44^ is incorporated into the loop substructure of MBA. Treatment of the MBA with ATP leads to a mixture of open-chain MBA, hairpin MBA, unbound ATP and the ATP/MBA complex (see step 2). The exact relative population of these species will be determined by the magnitude of the equilibrium constants *K*_1_ and *K*_2_. *K*_1_ becomes larger with increasing the hydrogen bonding interactions in the MBA stem substructure. *K*_2_ is determined by the ATP–aptamer interaction, which can be made nearly constant for all MBAs by using the same oligonucleotide sequence. Consider now step 3. Four types of electrostatic complexes may form immediately after CPE addition, including: open-chain MBA/CPE (1), hairpin MBA/CPE (2), a triplex of ATP/MBA/CPE (3) and ATP/CPE. However, a final equilibrium state will develop over time through opening of the hairpin structures by CPE. For instance, disruption of species 2 generates open-chain MBA/CPE, which in Scheme 2 is illustrated as 1′ to aid in subsequent discussion. Similarly, opening of species 3 yields 1′′. These two processes are contained in the red box of step 3. Greater FRET PL intensity and FRET ratio due to the higher population of open-chain MBA/CPE would be expected in the absence of ATP (blue box in step 3). After treatment with ATP, the resulting FRET signal comes from the open-chain MBA/CPE complex, *i.e.* the sum total of species, 1, 1′ and 1′′, in step 3, where the concentration of 1 is negligible with increasing stem stability and the total concentration of the emissive open-chain MBA/CPE is inversely proportional to [ATP].

**Fig. 1:**
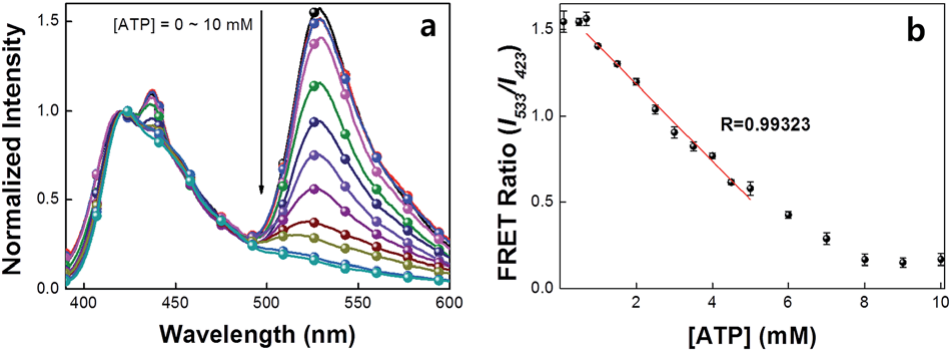
(a) Normalized FRET PL spectra and (b) detection range of MBA1/**PPFP-Br**. FRET spectra were obtained by exciting at 380 nm. [**PPFP-Br**] = 1.0 × 10^–6^ M, [MBA1] = 2.0 × 10^–8^ M. The FRET ratio shows linearity in the range of ∼mM ATP concentration range.

**Fig. 2:**
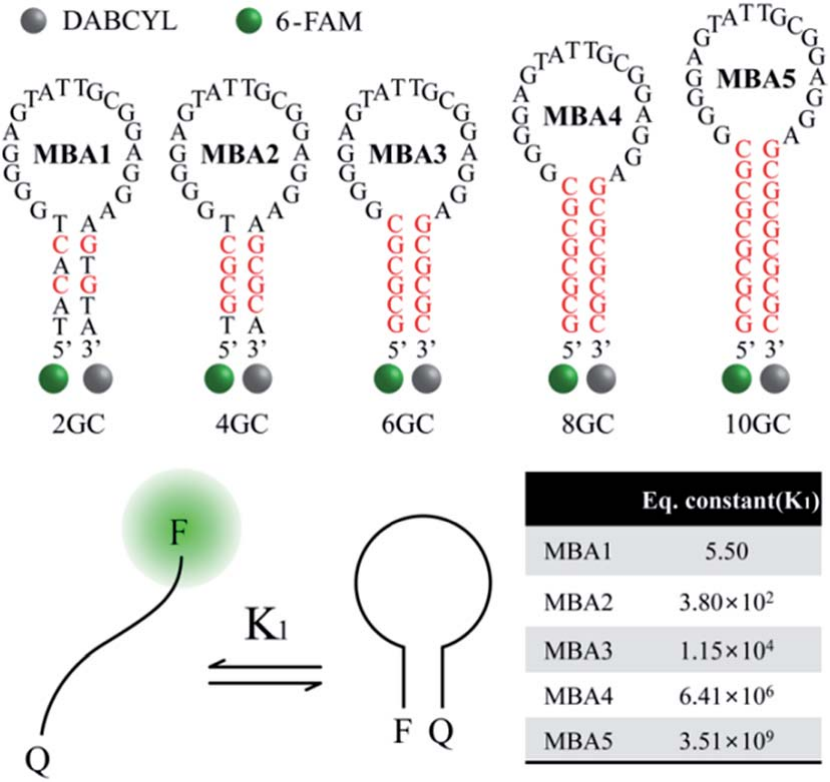
Molecular structures of the five MBAs used in the studies, together with calculated *K*_1_ values.

A series of experiments were carried out to validate Scheme 2. First, we examined the general basis of ATP/MBA/CPE interactions for providing a response toward [ATP]. We focused on MBA1 and the CPE, **PPFP-Br**. The molecular structure of MBA1 can be found in Fig. 2. Fig. 1 shows the normalized FRET PL spectra (upon excitation of **PPFP-Br** at 380 nm) of MBA1/**PPFP-Br** with changing [ATP] in Tris-HCl buffer ([**PPFP-Br**] = 1.0 × 10^–6^ M, [MBA1] = 2.0 × 10^–8^ M). One observes from Fig. 1 that the FRET-sensitized 6-FAM signal and the FRET ratio (*I*_533_/*I*_423_, where *I*_533_ is the PL intensity at 533 nm and *I*_423_ is the intensity at 423 nm) gradually decrease with increasing [ATP]. Under these conditions the ATP/MBA1 G-quadruplex concentration, namely species 3 in Scheme 2, increases and the resulting FRET-induced 6-FAM signal decreases. The FRET ratio shows a linear relationship in a range of [ATP] = 1.0 × 10^–3^ M to 7.1 × 10^–3^ M, from which a quantitative ATP detection calibration curve can be constructed for the MBA1/**PPFP-Br** combination, as shown in Fig. 1b. The detection range was determined by fitting a titration curve using the Hill equation, where the FRET ratio transits from 10% to 90% of its signal output.^45,46^ The LOD (3*σ*/slope, *σ* is the standard deviation from 4 independent measurements of the slope of the plot in Fig. 1b) was determined to be 5.7 × 10^–4^ M for MBA1/**PPFP-Br**. The data in Fig. 1 therefore provide a preliminary demonstration that the MBA1/**PPFP-Br** combination provides a quantitative analysis of [ATP] *via* the sequence of steps in Scheme 2.

### Modulation of detection characteristics by changing stem stability in MBAs

While the data in Fig. 1 demonstrate that the MBA1/**PPFP-Br** combination provides a successful detection of ATP, the detection sensitivity is limited (LOD = 0.57 mM) due to the weak binding (*K*_d_ = 1/*K*_2_ = μM–mM) between ATP and the aptamer sequence in MBA1.^35–39^ An efficient way to tune the sensitivity range by compensating the weak binding strength of ATP/aptamer is therefore required. Of particular practical relevance is that a limited detection range complicates, or in some instances precludes, the general use of biosensors in many real applications.

Fig. 2 shows the molecular structure of the five MBAs in our studies. They all contain the same ATP-specific aptamer sequence in the loop substructure but are anticipated to exhibit different stem stabilities due to the different number of G–C base pairs in the stem. In Scheme 2, *K*_2_ is not anticipated to be greatly influenced by the stem stability. This same binding affinity for all MBAs was identified by a series of experiments described below. MBA1, 2, 3, 4 and 5 have two, four, six, eight and ten G–C hydrogen bonds in the stem part, respectively. The equilibrium constant (*K*_1_, step 1) for the conformational transformation between the open-chain and hairpin structures was calculated to be *K*_1_ = 5.5 for MBA1, 3.8 × 10^2^ for MBA2, 1.2 × 10^4^ for MBA3, 6.4 × 10^6^ for MBA4 and 3.5 × 10^9^ for MBA5 (Table S1†).^47,48^ With increasing stem strength, the equilibrium is further shifted to the formation of the hairpin structure. Despite these different *K*_1_ values, all MBAs show a decreased 6-FAM emission by DABCYL when compared to analogous non-hairpin oligonucleotide sequences.

**Fig. 3:**
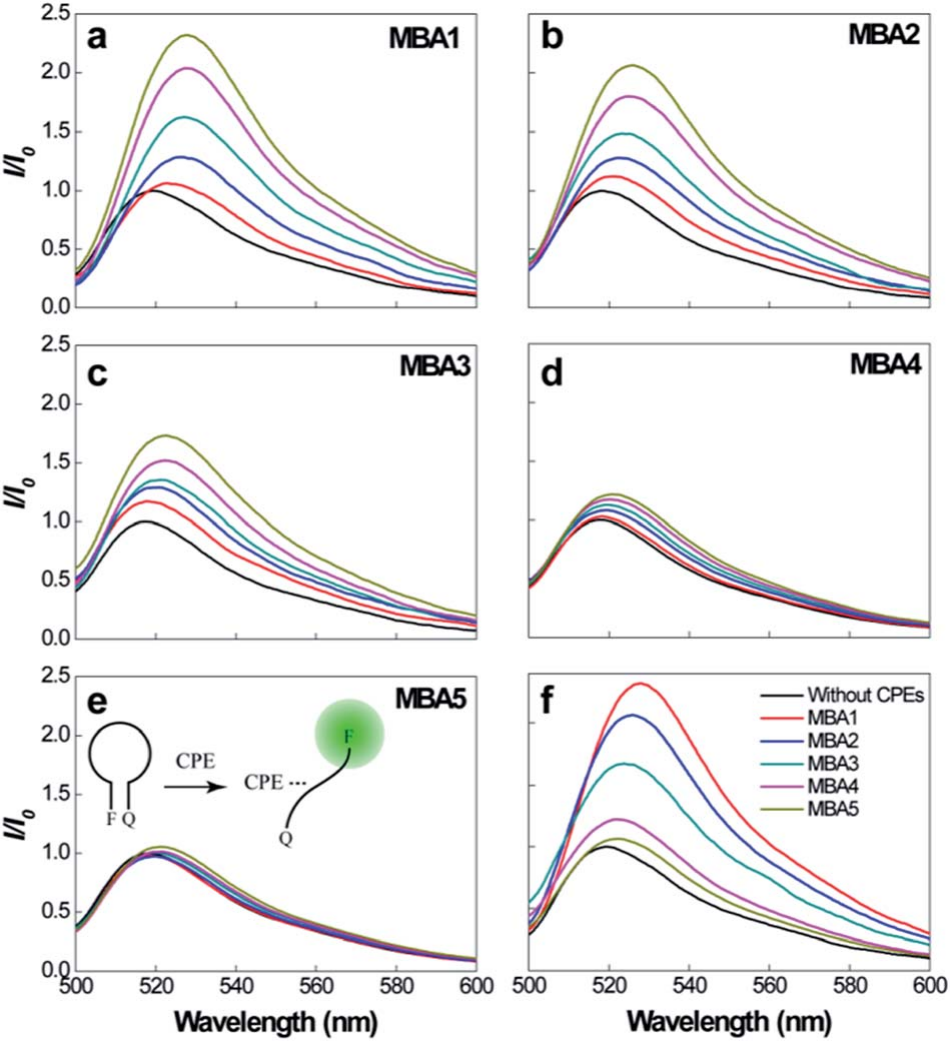
(a)–(e) PL spectra of MBA1–5 (2.0 × 10^–8^ M) upon direct excitation of 6-FAM at 490 nm, with increasing [**PPFP-Br**] = 0–1.0 × 10^–6^ M. (f) Comparison of PL signals of different MBAs with [**PPFP-Br**] = 1.0 × 10^–6^ M.

Prior to studying overall detection characteristics, the opening effect of the MBAs by **PPFP-Br** was investigated. These experiments relate to *K*_3_ in Scheme 2. Fig. 3 compares the 6-FAM PL signals of MBA1–5 upon direct excitation of 6-FAM at 490 nm, with and without the addition of **PPFP-Br**. With increasing [**PPFP-Br**] from 0 to 1.0 × 10^–6^ M, the 6-FAM signal gradually intensified. This increase is consistent with the opening of the hairpin structures by **PPFP-Br**.^34^ In the presence of 1 μM **PPFP-Br**, the 6-FAM signal intensity (*I*) was enhanced by approximately 2.3, 2.1, 1.7, 1.2 and 1.1 times for MBA1, 2, 3, 4 and 5, respectively, relative to that without **PPFP-Br** (*I*_0_). As expected, the 6-FAM emission recovery by **PPFP-Br** decreased gradually with increasing stem stability, indicating the decreased capability to disrupt the hairpin structure upon electrostatic and hydrophobic interactions with **PPFP-Br**. Another perspective is that as *K*_1_ increases, one obtains a decrease in *K*_3_.

**Fig. 4:**
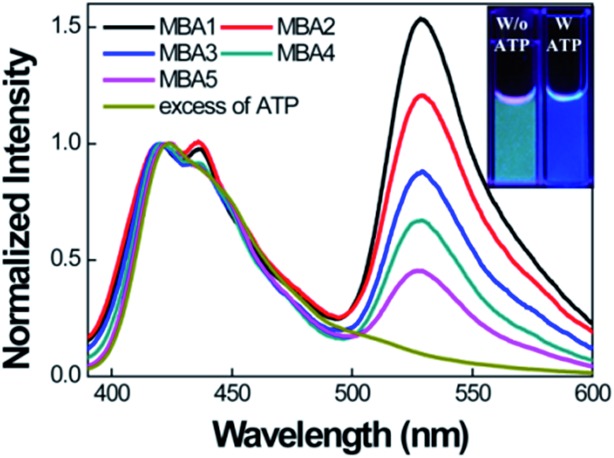
Normalized FRET-induced PL spectra of MBAs/**PPFP-Br** by exciting at 380 nm in the absence of ATP. [**PPFP-Br**] = 1.0 × 10^–6^ M, [MBA] = 2.0 × 10^–8^ M. The spectrum of MBA1/**PPFP-Br** with ATP (50 mM) is displayed together for comparison. Inset shows the photograph of the solutions with (blue) and without (greenish blue) ATP under UV illumination at 365 nm. Measurements were carried in 20 mM Tris-HCl buffer, pH 7.4.

We now examine how the various MBA structures lead to differences in 6-FAM FRET sensitization. Fig. 4 shows the PL spectra of MBAs/**PPFP-Br** in the absence and presence of ATP by exciting **PPFP-Br** at 380 nm ([**PPFP-Br**] = 1.0 × 10^–6^ M, [MBA] = 2.0 × 10^–8^ M). Looking at the results with MBA1, a strong FRET-induced PL signal from 6-FAM was measured in the absence of ATP. This observation reflects the formation of an open-chain MBA1/**PPFP-Br** electrostatic complex and illustrates the on-state, where the quenching action by DABCYL is circumvented. The addition of excess ATP (50 mM) leads to quenching of the FRET-induced 6-FAM signal due to formation of the ATP/MBA1 G-quadruplex (off-state, species 3 in Scheme 2). A clear change in the emission color from greenish blue to dark blue of the MBA1/**PPFP-Br** solution can also be observed by naked eye upon ATP addition (Fig. 4, inset). Examination of the PL profiles also reveals that with increasing stem stability (MBA1 → MBA5), the FRET ratio exhibits a gradual decrease due to the decreased opening of MBAs by **PPFP-Br** without ATP. In the absence of ATP, the FRET-induced PL intensity depends on the sum of the concentrations of open-chain/CPE complexes derived from the sum of species 1 and 1′ (blue box in Scheme 2), which are impacted by the stem stability. From a biosensor design, it is relevant that the FRET signal and on/off ratio can therefore be simply modulated by modification of MBA stem stability.

**Fig. 5:**
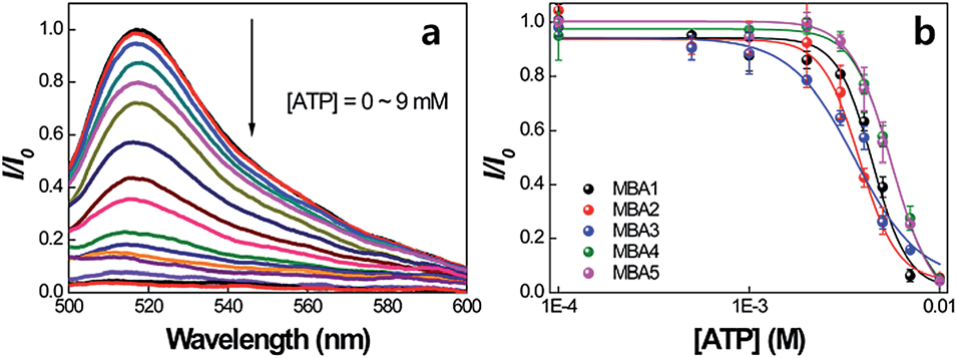
(a) PL spectra of MBA1 with increasing [ATP] = 0–9 mM, and (b) PL intensity ratio (*I*/*I*_0_) changes for MBAs as a function of [ATP]. *I* is the 6-FAM PL intensity at 517 nm and *I*_0_ is the original intensity without ATP. Emission spectra were obtained by exciting at 490 nm. [MBAs] = 2.0 × 10^–8^ M.

The idea that all MBAs should have similar ATP binding capabilities is now examined. Fig. 5a shows the PL signals of MBA1 (2.0 × 10^–8^ M) with changing [ATP] = 0–9 mM by exciting 6-FAM directly at 490 nm in the absence of **PPFP-Br**. The 6-FAM emission gradually decreases with increasing [ATP]. Such a decrease is consistent with further formation of the poorly emissive G-quadruplex structure possibly with a tightening of the internal structure such that the DABCYL quenching is accentuated. A plot of the intensity ratio *I*/*I*_0_ (where *I* is the 6-FAM PL intensity at 517 nm and *I*_0_ is the original intensity without ATP) against [ATP], as shown in Fig. 5b, provides a spectral response profile. When this analysis is carried out for the five MBA structures one observes that they provide a similar response. Specifically, the five MBA probes show a similar millimolar concentration detection range, as determined by the ATP concentration regime through which *I*/*I*_0_ transits from 10% to 90% of its signal output. These data allow us to estimate the dissociation constant,^21^ 1/*K*_2_ to be on the order of 3.2–5.4 mM and a LOD in the range of 1.8–2.7 mM for the five MBAs. The stem stability therefore does not appear to impact greatly the ATP/aptamer binding.

**Fig. 6:**
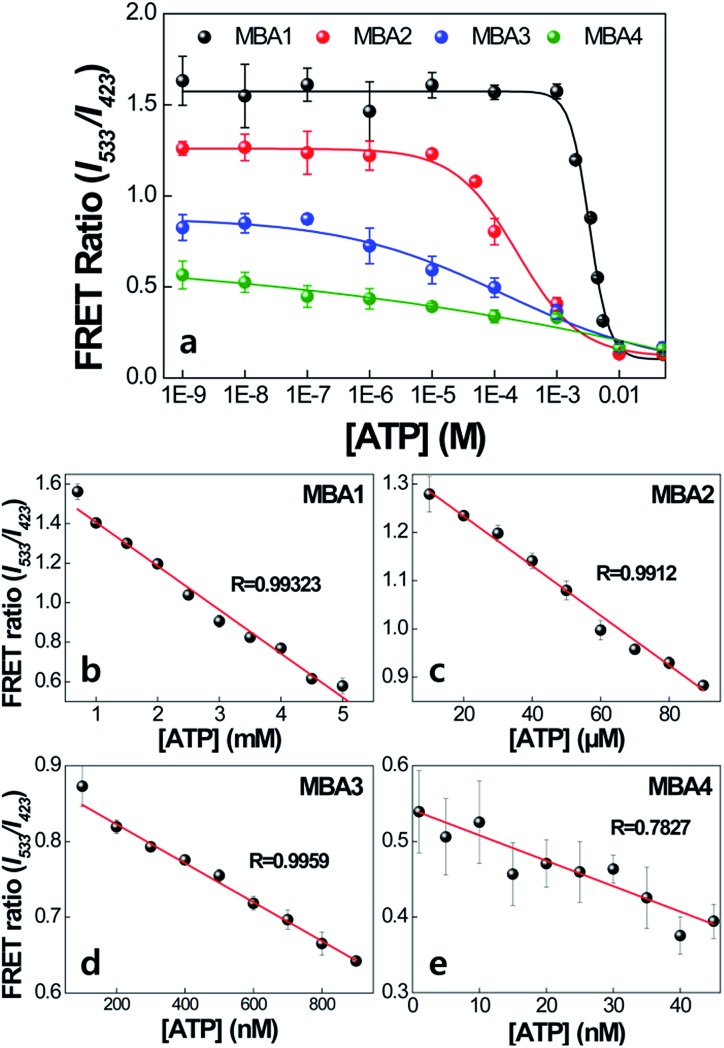
Detection range of MBA/**PPFP-Br** assays as a function of [ATP]. [**PPFP-Br**] = 1.0 × 10^–6^ M, [MBA] = 2.0 × 10^–8^ M. The data were obtained from four independent measurements and the error bars indicate the standard deviation.

The impact of the interactions with **PPFP-Br** to extend the detection range is discussed next. Fig. 6 compares the FRET ratio of MBA/**PPFP-Br** assays with changing [ATP] from 0 to 50 mM. These experiments were carried out by excitation of **PPFP-Br**. In the case of MBA1 with the weakest stem, the FRET ratio, shown with the black trace, increases with decreasing [ATP], is fully recovered at [ATP] = ∼5 × 10^–4^ M, and demonstrates a linear spectral response within a ∼mM [ATP] range. Comparison of the plots for MBA1–4 shows that increasing the stem stability leads to a substantial extension of the spectral response range. These ranges can cover from millimolar (1.0–7.1 × 10^–3^ M for MBA1/**PPFP-Br**), to micromolar (7.2 × 10^–6^–7.0 × 10^–3^ M for MBA2/**PPFP-Br**) and ultimately nanomolar (1.5 × 10^–8^–7.3 × 10^–3^ M for MBA3/**PPFP-Br** and 3.2 × 10^–9^–1.0 × 10^–3^ M for MBA4/**PPFP-Br**) ATP concentrations. LOD determinations for each MBA/**PPFP-Br** combination were done in a similar way to that in Fig. 1b using four independent measurements for each MBA and the results are shown in Fig. 6b–e. From these plots one can determine the LODs to be 5.7 × 10^–4^ M for MBA1, 1.2 × 10^–5^ M for MBA2, 3.1 × 10^–7^ M for MBA3 and 4.6 × 10^–8^ M for MBA4. It is worth noting that in the case of MBA5, we measured a similar trend but the signal to noise ratio was insufficient due to the weak FRET signal (see Fig. S1 in the ESI†).

The trends observed for the different MBAs may be rationalized by focusing on the two extreme cases, namely MBA1 and MBA4. Looking at the conditions in Fig. 6a with [ATP] = 0 M allows one to probe the situation where the difference in FRET signal is primarily determined by the ability of **PPFP-Br** to disrupt the hairpin structure (see blue box in Scheme 2). Under these conditions, the larger FRET ratio observed with MBA1 *vs.* MBA4 ultimately reflects the easier opening of the weaker stem. In the 10^–9^ to 10^–4^ M [ATP] range, there is no change in the FRET ratio with MBA1. Thus, the majority of species 3 is converted into 1′′ upon complexation with **PPFP-Br**. That the FRET ratio does change with MBA4 in this concentration range indicates a more resistant version of species 3 with the more stable stem structure. In other words, *K*_4_ is larger for MBA1 than MBA4. At the high concentration regime, as one reaches the millimolar range, even with the weakest stem (namely MBA1), one can shift the equilibrium to species 3 in which 6-FAM emission is effectively quenched within the G-quadruplex structure. MBA2 and MBA3 provide intermediate examples of the stability continuum and create the opportunity to examine intermediate concentration ranges. Thus, the differences in concentration sensitivity profiles most reasonably trace their origins to the ability of **PPFP-Br** to disturb species 3, the stability of which is reasonably determined to a large extent by *K*_1_. It is noted that as *K*_1_ increases, *K*_3_ and *K*_4_ decrease, influencing the [ATP] range showing spectral responses.

The conformational change of MBAs with/without ATP and **PPFP-Br** was also studied by measuring the circular dichroism (CD) spectra (Fig. S2†). The characteristic absorption bands of the quadruplex (ATP/MBA1) at 265 and 295 nm were observed in the presence of ATP ([MBA1] = 1.0 × 10^–6^ M, [ATP] = 5.0 × 10^–2^ M). Upon addition of **PPFP-Br** ([**PPFP-Br**] = 5.0 × 10^–5^ M), the peak intensity of the quadruplex (ATP/MBA1) decreased, indicating a partial breakup of the G-quadruplex structure by **PPFP-Br** (structural transformation into the open-chain form). The CD data also support the weak binding affinity between ATP and the aptamer. However, almost identical CD spectra were obtained for ATP/MBA3 in the presence and absence of **PPFP-Br**, suggesting the tighter folded G-quadruplex formation relative to ATP/MBA1. This emphasizes the role of stem strength to compensate the weak binding affinity between the aptamer and ATP for fine-tuning the sensor characteristics.

**Fig. 7:**
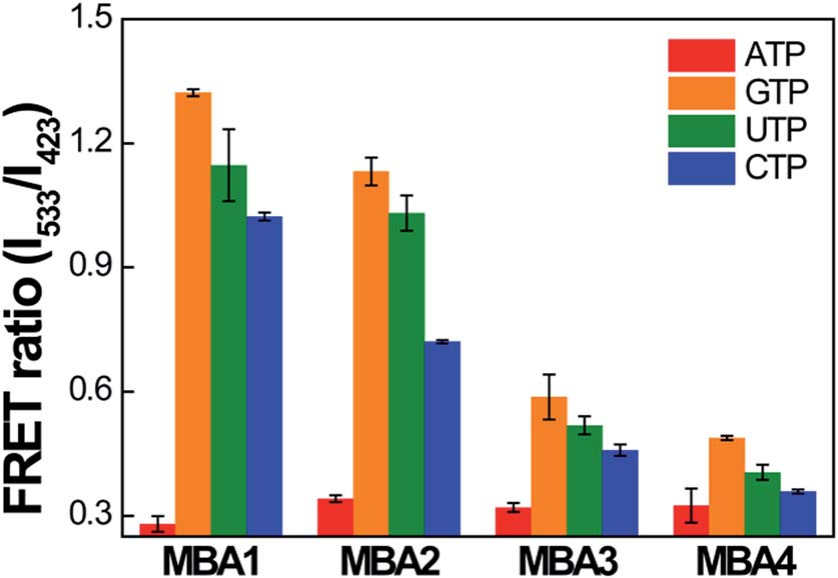
Selectivity data for MBA/**PPFP-Br** assays. FRET ratio (*I*_533_/*I*_423_) was compared in the presence of ATP and its analogues. [MBA] = 2.0 × 10^–8^ M, [**PPFP-Br**] = 1.0 × 10^–6^ M, [ATP analogues] = 5 mM. The data were obtained from four independent measurements and the error bars indicate the standard deviation.

The selectivity data of the MBA- and MBA/CPE-based ATP assays were measured against three ATP analogues, guanosine-5′-triphosphate (GTP), cytidine-5′-triphosphate (CTP) and uridine-5′-triphosphate (UTP). The PL signals of MBA1–5 (2.0 × 10^–8^ M) were measured in the presence of GTP, CTP and UTP (5 mM) by exciting 6-FAM directly at 490 nm without **PPFP-Br**. Because the ATP-specific aptamer recognizes the nucleoside unit of ATP, the binding affinity for other molecules is expected to be much weaker than ATP. The intensity ratio *I*/*I*_0_ (where *I* is the 6-FAM PL intensity at 517 nm and *I*_0_ is the original intensity without analytes) was compared with that in the presence of ATP, where the most serious PL quenching was measured with ATP for all MBA probes (Fig. S4†), suggesting remarkable selectivity against ATP analogues for all MBA-based assays. With regard to the MBA/CPE-based assays, the FRET spectra of MBA1–4/**PPFP-Br** were measured in the presence of ATP analogues (5 mM). In Fig. 7, the FRET ratio was compared for all MBA/**PPFP-Br** bioassays in the presence of ATP and ATP analogues. Although a substantial decrease in the FRET signal was observed with CTP, the smallest FRET ratio was measured in the presence of ATP, suggesting a clear selectivity for ATP. With increasing the stem stability in MBAs, the FRET signal (on state without analytes) decreases with decreased on/off ratio, resulting in the deteriorated selectivity. The MBAs with the higher stem strength enhanced the detection sensitivity with the extended dynamic range with the sacrificed signal on/off ratio, where a clear trade-off relationship was observed.

We also measured the FRET PL spectra of MBA1/**PPFP-Br** in 20 mM Tris-HCl buffer (pH 7.4) with changing salts. The ions such as K^+^, Mg^2+^ and Ca^2+^ can influence the G-quadruplex folding/unfolding and/or MBA's conformational change. Fig. S5† shows the FRET signal in the presence of 100 mM KCl, MgCl_2_, CaCl_2_ and all ions together. When compared to the case with NaCl, the FRET ratio (without ATP) slightly decreases in the presence of these ions, indicting the hairpin structure is more stabilized with less efficient opening by CPEs. However, the FRET signal is clearly quenched upon addition of ATP for all cases, suggesting that the MBA/CPE sensory system can respond to the presence of ATP, even in the presence of all K^+^, Mg^2+^ and Ca^2+^ ions together.

## Conclusions

In summary, we report how to combine synthetic CPEs and MBAs to create biosensor platforms that can be tuned to cover concentration ranges that extend beyond those determined by the specific aptamer-target binding constant. We also studied in detail the principal factors which govern the detection range tuning mechanism. In our specific example, the cationic **PPFP-Br** was combined with a range of MBA structures that maintain the same ATP recognition sequence but are modified by the stem composition so to exhibit different structural stabilities. In this MBA/CPE assay (Scheme 2), *K*_2_ is assumed to be nearly invariant because all MBAs have the same aptamer sequence in the loop, indicating no change in ATP binding affinity. The resulting FRET-sensitized signal depends on the differential opening of folded ATP/MBA G-quadruplexes by **PPFP-Br**, where the both ATP/aptamer binding (*K*_2_) and stem interactions (*K*_1_) compete with the MBA/**PPFP-Br** interaction. The equilibrium constant *K*_4_ decreases with increasing *K*_1_. With increasing stem stability, the folded ATP/MBA quadruplex structure becomes resistant to opening by **PPFP-Br**, even when [ATP] is low, which improves the limit of detection and broadens detection range.

From a practical perspective, the limit of detection was successfully modulated from millimolar (MBA1) to micromolar (MBA2), and further extended to nanomolar concentrations of ATP (MBA3 and MBA4). We note that [ATP] in living systems varies depending on the organelle and environment, typically 1–10 mM in cells and ∼1 μM in blood. The lower concentration level (∼nM) of human plasma ATP has also been reported.^49,50^ To cover the wide range of target concentrations found in a human body, a range-tunable detection strategy such as that provided by the combination of plots in Fig. 6 would be highly desirable.^22,51^ This strategy has the potential to be extended to other chemical- and biological-assays with low target binding affinity and is likely to be made functional by a range of other cationic CPE structures.

## Experimental

### General considerations

All chemicals were purchased from Aldrich Chemical Co. and used as received unless otherwise mentioned. High pressure liquid chromatography (HPLC)-purified molecular beacon aptamers labeled with 6-FAM and DABCYL with 30–38 bases (MBA1: 5′-6-FAM-TACA CTGG GGAG TATT GCGG AGGA AGTG TA-DABCYL-3′, MBA2: 5′-6-FAM-TGCG CTGG GGAG TATT GCGG AGGA AGCG CA-DABCYL-3′, MBA3: 5′-6-FAM-GCGC GCGG GGAG TATT GCGG AGGA GCGC GC-DABCYL-3′, MBA4: 5′-6-FAM-GCGC GCGC GGGG AGTA TTGC GGAG GAGC GCGC GC-DABCYL-3′, MBA5: 5′-6-FAM-GCGC GCGC GCGG GGAG TATT GCGG AGGA GCGC GCGC GC-DABCYL-3′) were obtained from Sigma-Genosys.

UV/vis absorption spectra were measured by using a Jasco (V-630) spectrophotometer. The photoluminescence spectra were obtained on a Jasco (FP-6500) spectrofluorometer with a Xenon lamp excitation source, using 90° angle detection for the solution samples. The fluorescence quantum yield was measured relative to a standard fluorescein solution in water at pH = 11. The circular dichroism spectra were obtained using a Jasco (J-715) 202 circular dichroism spectrometer.

### Synthesis and characterization of CPE (**PPFP-Br**)

The cationic CPE, poly[(9,9′-bis(4-(6-*N*,*N*,*N*-trimethylammoniumhexyloxy)phenyl)fluorene-2,7-diyl)-*alt*-1,4-phenylene dibromide] (**PPFP-Br**) was synthesized following previously reported procedures.^52,53^ The number average molecular weight of **PPFP-Br** was determined to be Mn = 16 000 g mol^–1^ (polydispersity index = 2.7) by examination of the neutral precursor by gel permeation chromatography relative to polystyrene standards. The absorption and PL maxima of **PPFP-Br** were measured at *λ*_abs_ = 375 nm and *λ*_PL_ = 423 nm, respectively, in a 20 mM Tris-HCl buffer solution with 100 mM NaCl. **PPFP-Br** shows a PL quantum efficiency of ∼46% in this buffer. The emission of **PPFP-Br** shows a good spectral overlap with the absorption of 6-FAM (*λ*_abs_ = 490 nm) as the FRET acceptor (Fig. S3 in the ESI†).

### ATP detection assay protocols

PL experiments were performed in 20 mM Tris-HCl buffer (pH = 7.4) containing 100 mM NaCl. A stock solution (10^–5^ M) of each MBA was prepared in deionized water. Each MBA stock solution (4 μL) was added to 2 mL buffer and the resulting solutions were incubated around the melting temperatures of MBAs (at 65 °C for MBA1, 75 °C for MBA2, 80 °C for MBA3 and 90 °C for MBA4 and 5, respectively) for 20 min with changing [ATP]. The annealed solution was cooled down slowly to room temperature for 1.5 h **PPFP-Br** (20 μL, 10^–4^ M in water/dimethylsulfoxide (3 vol%)) was then added to the above solution and the PL spectra were measured by exciting either the polymer or 6-FAM. The final concentration of MBA and **PPFP-Br** in the assay system was 2.0 × 10^–8^ M and 1.0 × 10^–6^ M, respectively. A charge ratio ([+]/[–], [+] in **PPFP-Br** : [–] in MBA) of ∼3 was chosen for measuring PL spectra.

For the opening test of MBAs by **PPFP-Br**, the MBA solution ([MBA] = 2.0 × 10^–8^ M) in Tris-HCl buffer was annealed around its melting temperature for 20 min and cooled down to room temperature over a period of 1.5 h to achieve the equilibrium state. The PL spectra were measured by exciting 6-FAM at 490 nm with increasing [**PPFP-Br**] = 0–1.0 × 10^–6^ M at room temperature by successive addition of 4 μL of aq. stock solution (10^–4^ M) of **PPFP-Br**.
