# Supplementary material for: Principal factors that determine the extension of detection range in molecular beacon aptamer/conjugated polyelectrolyte bioassays
Source: Chem Sci. 2015 Jan 7;6(3):1887–94. doi: 10.1039/c4sc03258f (PMC5494547; doi:10.1039/c4sc03258f)
Supplement: Supplementary file 1 [file SC-006-C4SC03258F-s001.pdf]

SUPPORTING INFORMATION

Principal Factors that Determine the Extension of Detection Range in Molecular Beacon Aptamer/ Conjugated Polyelectrolyte Bioassays

Ji-Eun Jeong,[a] Boram Kim,[a] Shinjae Woo,[a] Sungu Hwang,[b]
Guillermo C. Bazan,*[c] and Han Young Woo*[a]

[a] Department of Nanofusion Engineering, Department of Cogno-Mechatronics Engineering,
Pusan National University, Miryang, Gyeongsangnam-do 627-706, Republic of Korea. E-mail: hywoo@pusan.ac.kr

[b] Department of Nanomechatronics Engineering,
Pusan National University, Miryang, Gyeongsangnam-do 627-706, Republic of Korea

[c] Department of Chemistry & Biochemistry, University of California, Santa Barbara,
California 93106, United States. E-mail: bazan@chem.ucsb.edu

Table S1. Equilibrium constants for conformational transformation for MBAs

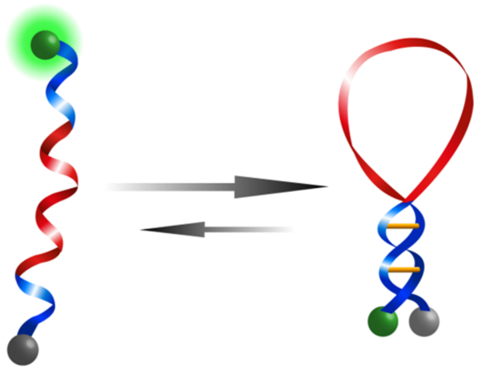

	Aptamer sequence (5′ – 3′)	ΔG°	Eq. constant (K)
MBA1	[6-FAM] TACA CTGG GGAG TATT GCGG AGGA AGTG TA [DABCYL]	-1.1 kcal/mol	5.5
MBA2	[6-FAM] TGCG CTGG GGAG TATT GCGG AGGA AGCG CA [DABCYL]	-3.7 kcal/mol	3.8×10^2
MBA3	[6-FAM] GCGC GCGG GGAG TATT GCGG AGGA GCGC GC [DABCYL]	-5.8 kcal/mol	1.2×10^4
MBA4	[6-FAM] GCGC GCGC GGGG AGTA TTGC GGAG GAGC GCGC GC [DABCYL]	-9.7 kcal/mol	6.4×10^6
MBA5	[6-FAM] GCGC GCGC GCGG GGAG TATT GCGG AGGA GCGC GCGC GC [DABCYL]	-13.5 kcal/mol	3.5×10^9

*** Calculation of Gibbs free energy**

http://mfold.rna.albany.edu

The equilibrium constants for conformational change of MBAs were obtained at the above website where the minimum Gibbs free energy (G) and standard Gibbs free energy change, ΔG^0, were calculated for folding of nucleic acids with particular base sequence (37 ^{o}C, $[Na^+]$ = 100 mM and $[Mg^{2+}]$ = 0 M).

*** Equilibrium Constant (K)**

$\Delta G = \Delta G^\circ + RT\ln K$

In equilibrium state, $\Delta G = 0$

$\therefore \Delta G^\circ = -RT\ln K$

$\ln K = \Delta G^\circ / -RT$

$K = e^{(\Delta G^\circ/-RT)}$

Where R is gas constant (1.9863 cal/k·mol), T is temperature (310.15 K) and G is Gibbs free energy.

 This journal is © The Royal Society of Chemistry 2012

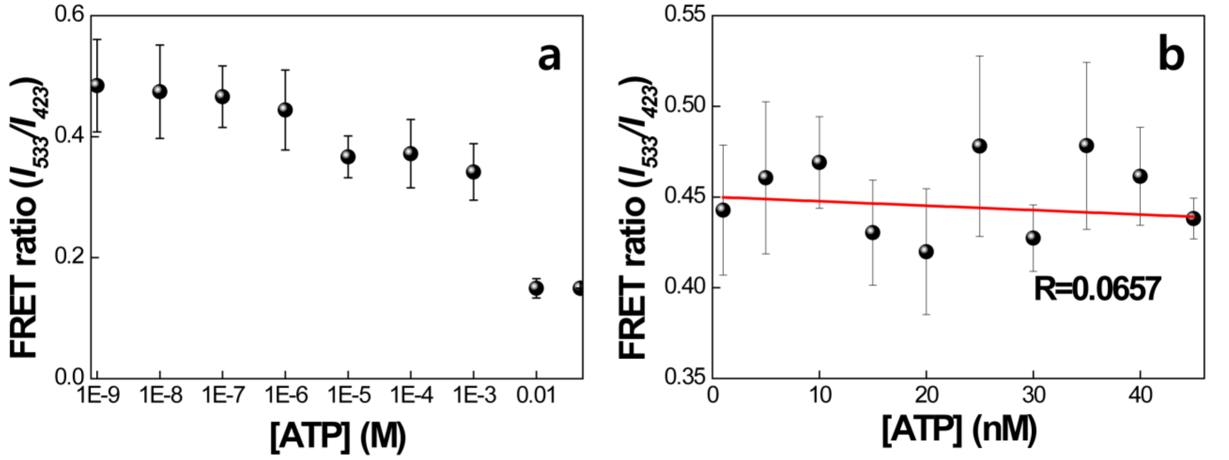

Figure S1. FRET ratio of MBA5/**PPFP-Br** assay with changing [ATP]. Detection range and LOD could not be determined due to insufficient signal to noise ratio. [**PPFP-Br**] = 1.0 × 10^{-6} M, [MBA] = 2.0 × 10^{-8} M. The data were obtained from four independent measurements and the error bars indicate the standard deviation.

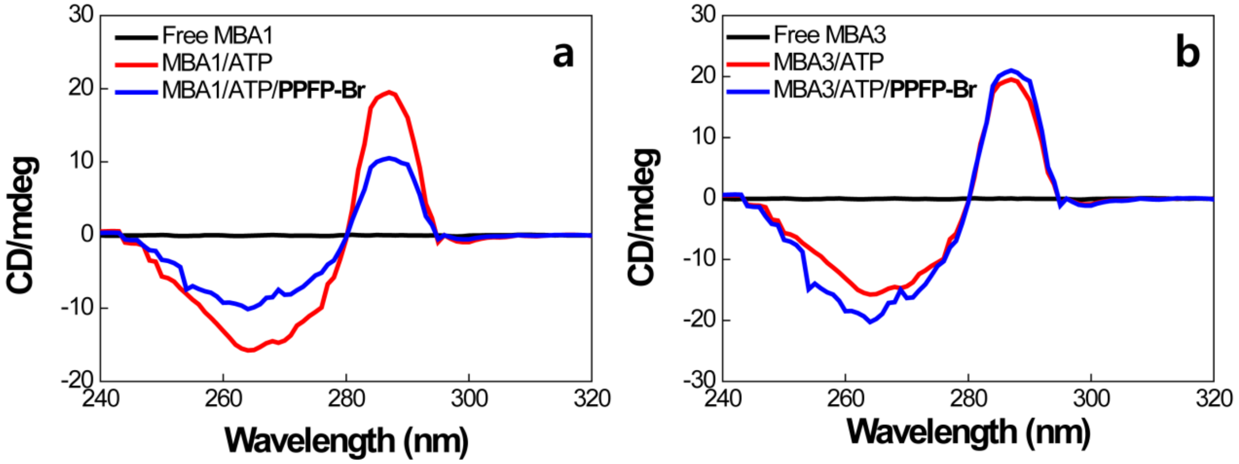

Figure S2. Circular dichroism spectra of (a) MBA1 and (b) MBA3 in the presence and absence of ATP and **PPFP-Br**. [MBAs] = 1.0 × 10^{-6} M, [ATP] = 5.0 × 10^{-2} M, [**PPFP-Br**] = 5.0 × 10^{-5} M.

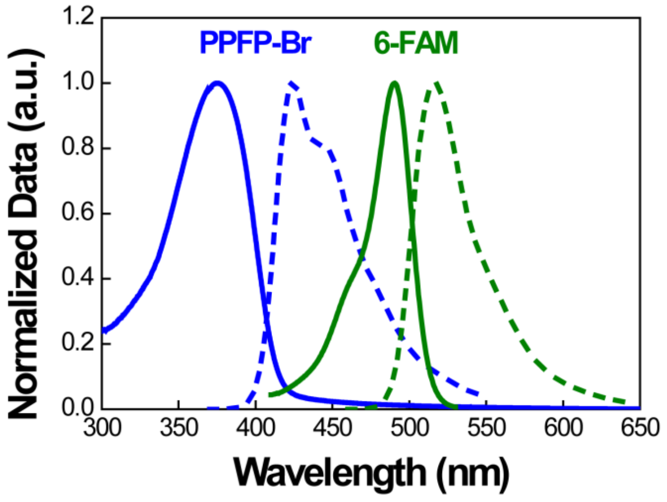

Figure S3. UV/Vis and PL spectra of **PPFP-Br** and 6-FAM in 20 mM Tris buffer (pH 7.4) with [NaCl] = 100 mM as a salt.

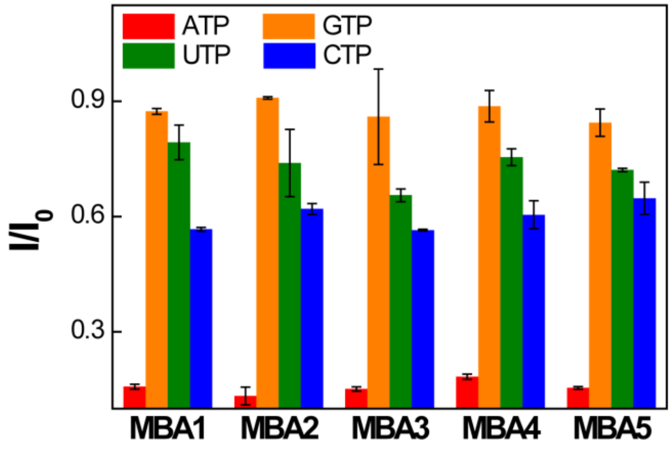

Figure S4. Selectivity data for MBA-based ATP detection without **PPFP-Br**. [ATP analogues] = 5 mM, [MBA] = 2.0 × 10^{-8} M. The data were obtained from four independent measurements and the error bars indicate the standard deviation.

 This journal is © The Royal Society of Chemistry 2012

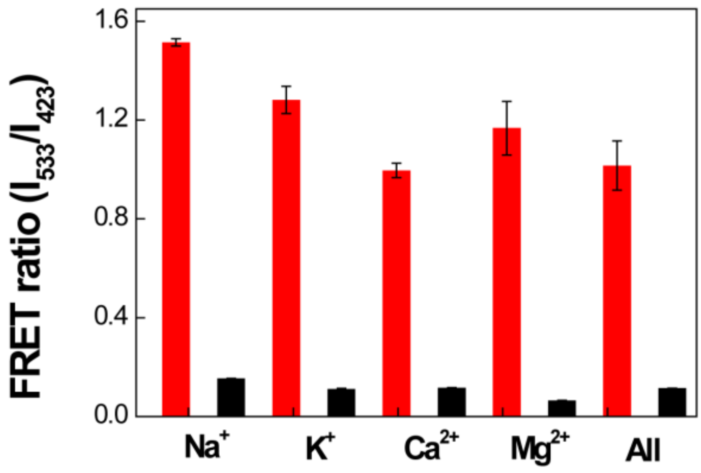

Figure S5. FRET ratio (I_{533}/I_{423}) of MBA1/**PPFP-Br** in the presence of different salts. [KCl, $MgCl_2$, or $CaCl_2$] = 100 mM, [**PPFP-Br**] = 1.0 × 10^{-6} M, [MBA1] = 2.0 × 10^{-8} M, [ATP] = 0 or 20 mM (red: without ATP, black: with ATP). The data were obtained from four independent measurements and the error bars indicate the standard deviation.
